# Short segment rib resection to mitigate risk of pleural violation during retropleural lateral thoracic interbody fusion

**DOI:** 10.3171/2022.3.FOCVID21138

**Published:** 2022-07-01

**Authors:** Bo Li, Gregory A. Kuzmik, Saman Shabani, Nitin Agarwal, Alysha Jamieson, Thomas Wozny, Simon Ammanuel, Praveen V. Mummaneni, Dean Chou

**Affiliations:** 1 Department of Neurosurgery, University of California, San Francisco, California; and; 2 Department of Clinic of Spine Center, Xinhua Hospital, Shanghai Jiaotong University School of Medicine, Shanghai, China

**Keywords:** lateral thoracic surgery, minimally invasive surgery, spine surgery, retropleural approach

## Abstract

It can be difficult to avoid violating the pleura during the retropleural approach to the thoracolumbar spine. In this video, the authors resect a short segment of rib to allow more room for pleural dissection during a minimally invasive (MIS) lateral retropleural approach. After a lateral MIS skin incision, the rib is dissected and removed, clearly identifying the retropleural space. The curvature of the rib can then be followed, decreasing the risk of pleural violation. The pleura can then be mobilized ventrally until the spine is accessed. Managing the diaphragm is also illustrated by separating the fibers without a traditional cut through the muscle.

The video can be found here: https://stream.cadmore.media/r10.3171/2022.3.FOCVID21138

## Transcript

This is a short-segment rib resection to mitigate risk of pleural violation during retropleural lateral thoracic interbody fusion.

### 0:29

The procedural rationale is that without removing the rib, it can be difficult to stay retropleural during lateral thoracic approach. This procedure still maintains the minimally invasive nature and is less morbid than an open thoracotomy.^1–3^ The benefits include no chest tube, clear visualization of the retropleural plane, a larger working channel to dissect the pleura than an intercostal-only approach and obtain rib graft for fusion. The risks include intercostal neuralgia, pleural violation despite dissection, and potential pneumothorax.

### 1:05

In terms of the alternatives and why they were not chosen, a true intercostal approach without rib resection can be very difficult to stay lateral to the spine and dissect completely retropleurally and dissect the pleura off the rib without a violation. The small intercostal window prevents a larger retractor opening, and there’s potentially a higher risk of intercostal neuralgia from the retractor pressing against the nerve and the rib.^4^ An open thoracotomy can be more morbid, requires a chest tube, and often requires that the lung is deflated intraoperatively.^5^

### 1:40

In terms of positioning, the patient is positioned in the lateral decubitus position. The necessary equipment includes navigation or fluoroscopy, rib dissection and resection tools, minimally invasive lateral spine retractor, and a lateral interbody fusion system.

### 1:58

The key steps of the procedure are to identity the disc space with fluoroscopy or navigation, making the skin incision over the rib, dissecting the pleura away from the rib, and excising approximately 2 inches of rib. Following the pleura dorsally along the rib until the spine is identified. Identify the rib head and disc space, confirming with imaging, and dock the minimally invasive retractor after identifying the disc space. Subsequently, the lateral interbody thoracolumbar fusion can be performed.

### 2:33

At the thoracolumbar junction, sometimes the rib cage can be blocking access to the upper lumbar spine, such as L1–2 or even L2–3. At this point, the retropleural approach can be performed going through the diaphragm to access the retroperitoneal lumbar spine. Because the working portal is so small, it is often impractical to cut the diaphragm as in an open surgery, which leaves a cuff of tissue against the chest wall. Instead, one option is to bluntly dissect through the diaphragm fibers in the direction of the fibers to expose the lumbar spine through the chest cavity.

### 3:10

This is an artist illustration demonstrating the traditional minimally invasive approach without resecting the rib. You can see that the trajectory is directly lateral from the lateral aspect of the rib cage toward the spine, but there is a significant dorsal component of pleura that is attached to the rib and very difficult to access with this approach. This can result in pneumothorax by resecting a small piece of rib. Retropleural dissection can be performed first, dorsally against the rib cage. Gently dissecting the pleura off the rib, as the rib is tracked down toward the spine.

### 3:51

This demonstrates the incisional planning with the patient in the right lateral decubitus position and the surgeon standing on the abdominal side. Using navigation, the interspace can be identified and the rib that needs to be resected can also be identified. As a general rule, the rib is resected posteriorly in order to dissect the pleura off the thoracic rib cage. If the interspace falls between two ribs, generally the inferior rib is removed in order to allow for more posterior dissection. Usually, one rib resection will allow for two interspaces, and if more spaces need to be performed, a separate incision is then performed.

### 4:30

Here, you can see in the upper left the rib overlying the spine, and this is the navigational probe that is used to plan the incision over the rib in order to facilitate a retropleural approach. Using the navigation, the appropriate rib could then be resected and a posterior dissection can then be performed down into the thoracic spine.

### 4:53

The first case is a 70-year-old woman who presents with severe back and left leg pain.

### 4:58

Here are the standing AP and lateral long films, which demonstrate the position of the rib cage over the upper lumbar and lower thoracic spine. The red arrow demonstrates the trajectory and the need to go through the rib cage to access the lower thoracic and upper lumbar spine.

### 5:20

Here you can see the patient’s MRI demonstrating lateral recess stenosis, but no severe central stenosis.

### 5:26

Here you can see the orientation of the surgical field. A traditional minimally invasive opening is performed in the standard manner. After the skin and fascia have been dissected, there is chest wall muscle that is present over the rib. This can be dissected with electrocautery. Extreme care must be taken to ensure that the intercostal space is not violated with the electrocautery, and the rib is continually palpated to ensure that the dissection is over the actual rib itself. The cephalad and caudad portions of the rib and the musculature can be dissected using electrocautery, but care must be taken so that violation of the pleura does not happen with the electrocautery. A small curette can then be used to dissect the pleura off the rib, and this can be done in the cephalad and in the caudad portions of the rib. The intercostal neurovascular bundle should usually be dissected away. In this case, a Kerrison punch is used to resect the rib. After dissection away from the pleura, and a very small piece of rib here is resected to increase the size of the window and access the retropleural space. The rib is then subsequently removed and can be used for fusion. The retropleural space is then dissected using a Penfield no. 1 against the remaining rib, and the plane can be subsequently opened using the Penfield no. 1 all the way down to the spine. You can see that we are dissecting all the way down following the rib until we reach the thoracic spine. The disc is identified, and the navigational probe is placed, and the minimally invasive dilators are used to subsequently allow the retractor to be placed. After this has been done, the discectomy and interbody fusion are performed in a standard manner.

### 7:43

Here are the postoperative radiographs of the lateral interbody fusion stage, demonstrating the trajectory through which the interbody grafts were placed.

### 7:55

The second case is a 69-year-old male who presents with severe back and left leg pain. He has an inability to stand erect, and he is leaning forward.

### 8:05

Here you can see the patient’s standing AP and lateral scoliosis x-rays. A minimally invasive scoliosis surgery was planned, and in order to access the lower thoracic spine—you can see that where the red arrow is—the rib cage needs to be traversed.

### 8:22

Here you can see the MRI of the patient.

### 8:25

Here you can see the standard minimally invasive incision over the rib cage is made, and the rib is subsequently dissected using electrocautery. A curette is then used to initiate the dissection of the pleura off the rib, and here you can see the standard thoracotomy (Doyan dissector) being used, and a standard thoracotomy rib cutter being used to cut the rib. This is another option that can be used in order to remove the rib.

### 8:59

You can see the remnant of the rib there, and using a Penfield no. 1 dissector, this plane of the retropleural space can be followed. In this example, we will demonstrate the management of the diaphragm. After dissection down to the thoracic spine, the diaphragm will be in the way at T11–12. And here you can see the dissection to the rib head. The diaphragm can then be dissected in the direction of its fibers. And here you can see blunt dissection of the diaphragm in the direction of its fibers. And using a bipolar cautery, the edges are bipolared, and the disc is accessed with a small window through the diaphragm. After this has been done, the disc is accessed using a probe, and the minimally invasive dilators are placed and the standard lateral interbody fusion is performed.

### 10:08

Here you can see postoperative radiographs of the anterior stage demonstrating the interbody fusion through the rib cage.

## Disclosures

Dr. Agarwal reports other from Thieme Medical Publishers and Springer Nature, outside the submitted work. Dr. Mummaneni reports personal fees from DePuy Synthes, Globus, and Stryker, outside the submitted work; royalties from DePuy Synthes, Thieme Publishers, and Springer Publishers; and stockholder with Spinicity/ISD. Dr. Chou reports personal fees from Globus and Orthofix, outside the submitted work.

## Author Contributions

Primary surgeon: Chou. Assistant surgeon: Wozny. Editing and drafting the video and abstract: Shabani, Li, Kuzmik, Agarwal, Jamieson, Wozny, Ammanuel, Chou. Critically revising the work: Shabani, Li, Agarwal, Wozny, Ammanuel, Chou. Reviewed submitted version of the work: Shabani, Li, Kuzmik, Agarwal, Wozny, Ammanuel, Mummaneni, Chou. Approved the final version of the work on behalf of all authors: Shabani. Supervision: Chou.

## Supplemental Information

### Patient Informed Consent

The necessary patient informed consent was obtained in this study.
